# *Actinidia chinensis* Planch root extract inhibits cholesterol metabolism in hepatocellular carcinoma through upregulation of *PCSK9*

**DOI:** 10.18632/oncotarget.15010

**Published:** 2017-02-02

**Authors:** Mingyan He, Jiayun Hou, Lingyan Wang, Minghuan Zheng, Tingting Fang, Xiangdong Wang, Jinglin Xia

**Affiliations:** ^1^ Liver Cancer Institute, Zhongshan Hospital, Fudan University, Shanghai, China; ^2^ Clinical Science Institute, Zhongshan Hospital, Fudan University, Shanghai, China; ^3^ Minhang Hospital, Fudan University, Shanghai, China

**Keywords:** hepatocellular carcinoma, root of Actinidia chinensis, PCSK9, cholesterol metabolism

## Abstract

*Actinidia chinensis* Planch root extract (acRoots) is a traditional Chinese medicine with anti-tumor efficacy. To investigate the mechanisms responsible for this activity, we examined the effects of acRoots on cholesterol metabolism in hepatocellular carcinoma (HCC). mRNA chip analysis was used to identify the metabolic genes regulated by acRoots. The effects of acRoots on cholesterol synthesis and uptake were evaluated by measuring intracellular cholesterol levels and 3,3′-dioctadecylindocarbocyanine-labeled low-density lipoprotein (Dil-LDL) uptake. Expression of metabolic genes was analyzed using quantitative reverse transcription PCR, western blotting, and flow cytometry. acRoots reduced the viability of LM3 and HepG2 cells at 5 mg/mL and HL-7702 cells at 30 mg/mL. Gene expression profiling revealed that treatment with acRoots altered expression of genes involved in immune responses, inflammation, proliferation, cell cycle control, and metabolism. We also confirmed that acRoots enhances expression of *PCSK9*, which is important for cholesterol metabolism. This resulted in decreased *LDL receptor* expression, inhibition of LDL uptake by LM3 cells, decreased total intracellular cholesterol, and reduced proliferation. These effects were promoted by *PCSK9* overexpression and rescued by *PCSK9* knockdown. Our data demonstrate that acRoots is a novel anti-tumor agent that inhibits cholesterol metabolism though a *PCSK9*-mediated signaling pathway.

## INTRODUCTION

Hepatocellular carcinoma (HCC) is a malignant tumor of the liver. It is the third most common cause of cancer-related death worldwide [1, 2]. Asian countries (particularly China) account for 55% of all newly diagnosed HCC cases and more than 50% of HCC deaths worldwide [3]. The 5-year survival rate for HCC patients is 15% (localized, 28%; regional, 7%; and distant, 3%), and 80-90% of patients present with advanced and unresectable tumors at the time of diagnosis due to widespread intra- and extra-hepatic metastasis [4]. Thus, it is critical to elucidate the molecular mechanisms underlying HCC progression and develop novel treatment strategies. Several drugs have shown efficacy in HCC including the multi-kinase inhibitor Sorafenib (targets Raf, VEGFR, PDGFRβ, c-Kit, RET, and other kinases), which is approved for clinical use. However, the therapeutic limitation has also been demonstrated in several clinical trials [5, 6].

*Actinidia chinensis* Planch root extract (acRoots) is a traditional Chinese medicine that has been used for the treatment of various types of cancer including HCC [7]. The principal compounds are triterpenes, which exhibit remarkable anticancer effects in HCC [7–9]. Other compounds have also shown cytotoxic activity including the phenolic constituents and isomeric flavonoids [10, 11]. Although the anticancer effects of acRoots have been observed clinically, the mechanisms underlying the effects are not fully understood.

Many recent studies have described metabolic alterations in proliferating cancer cells [12]. Using a non-targeted metabolic profiling strategy based on liquid chromatography-mass spectrometry, Huang et al. demonstrated that the predominant metabolic alternations in HCC included increased glycolysis, gluconeogenesis, and β-oxidation with reduced tricarboxylic acid cycle and Δ-12 desaturase [13]. These metabolic alterations provide the nutrients and energy to support the uncontrolled proliferation of malignant cells. Therefore, therapeutics that target these metabolic pathways in cancer cells may be effective in HCC.

Triterpenes from *Alismatis rhizoma* were shown to have a lipid-lowering effect on mice with high-fat diet-induced hyperlipidemia [14]. Therefore, we hypothesized that acRoots could alter metabolic processes in HCC cells. Here, we show that acRoots inhibits cholesterol metabolism in HCC cell lines through upregulation of PCSK9.

## RESULTS

### acRoots inhibits proliferation and regulates metabolic pathways in HCC

**Figure 1:**
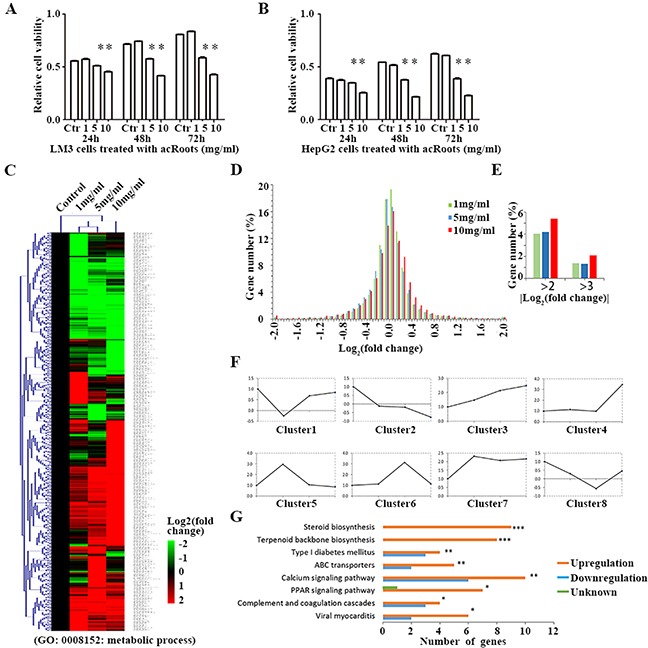
Metabolic gene profiling of LM3 cells treated with acRoots **A, B**. Proliferation ability of LM3 and HepG2cells after treatment with acRoots; **C**. Hierarchical clustering of all metabolic genes regulated by acRoots in LM3 cells. The genes are annotated to the term “metabolic process” (GO: 0008152) or its children in Gene Ontology; **D, E**. Distribution map of mRNA changes induced by a concentration gradient of acRoots; **F**. Eight classes based on the similarity of the drug concentration profiles: clusters 2 and 3, corresponding to acRoots-induced up- or down-regulation of genes in a dose-dependent manner; **G**. Enrichment of KEGG pathways in the metabolic gene profiling analysis was determined using the DAVID software. Benjamini-Hochberg adjusted p values are shown for each indicated bar (***p < 0.0001, **p < 0.05, *p < 0.1).

To assess the effects of acRoots on HCC cells, we treated human HCC cell lines (LM3 and HepG2), and normal liver cells (HL-7702) with various doses of acRoots and analyzed cell viability in response to treatment. CCK-8 assays revealed that acRoots treatment inhibited cell viability in a dose-dependent manner (Figure 1A, 1B, and Supplementary Figure 1A). These effects were observed at a dose of 5 mg/mL in LM3 and HepG2 cells, and at a dose of 30 mg/mL in HL-7702 cells. LM3 cells were selected for gene expression profiling. The results of mRNA profiling suggested that acRoots induced variations in the expression of genes involved in the immune response, inflammation, proliferation, cell cycle control, and metabolism in LM3 cells (Supplementary Figure 1B). We focused on genetic variations in metabolic genes annotated to the term “metabolic process” (GO:0008152) and related children in Gene Ontology (http://amigo1.geneontology.org/) (Supplementary Data sets 1 and 2). We quantified the expression of 711 metabolic genes in LM3 cells that showed at least a two-fold change in expression compared to the untreated control group after treatment with any concentration of acRoots (Figure 1C, Supplementary Data set 3). Distribution maps were plotted to show differences in mRNA levels induced by various doses of acRoots. These data indicated acRoots inhibited the expression of metabolic genes in a dose-dependent manner (Figure 1D and 1E). Hierarchical clustering analysis yielded eight clusters based on the similarity of the drug concentration profiles (Figure 1F). The Database for Annotation, Visualization, and Integrated Discovery (DAVID) software was then used to determine which Kyoto Encyclopedia of Genes and Genomes (KEGG) pathways were enriched [15, 16]. We found that several members of the steroid and terpenoid backbone biosynthesis pathways were significantly up-regulated in LM3 cells (Figure 1G, Supplementary Figure 1C and 1D), suggesting that acRoots altered steroid metabolism in HCC cells.

### Expression analysis of metabolic genes in LM3 cells

**Figure 2:**
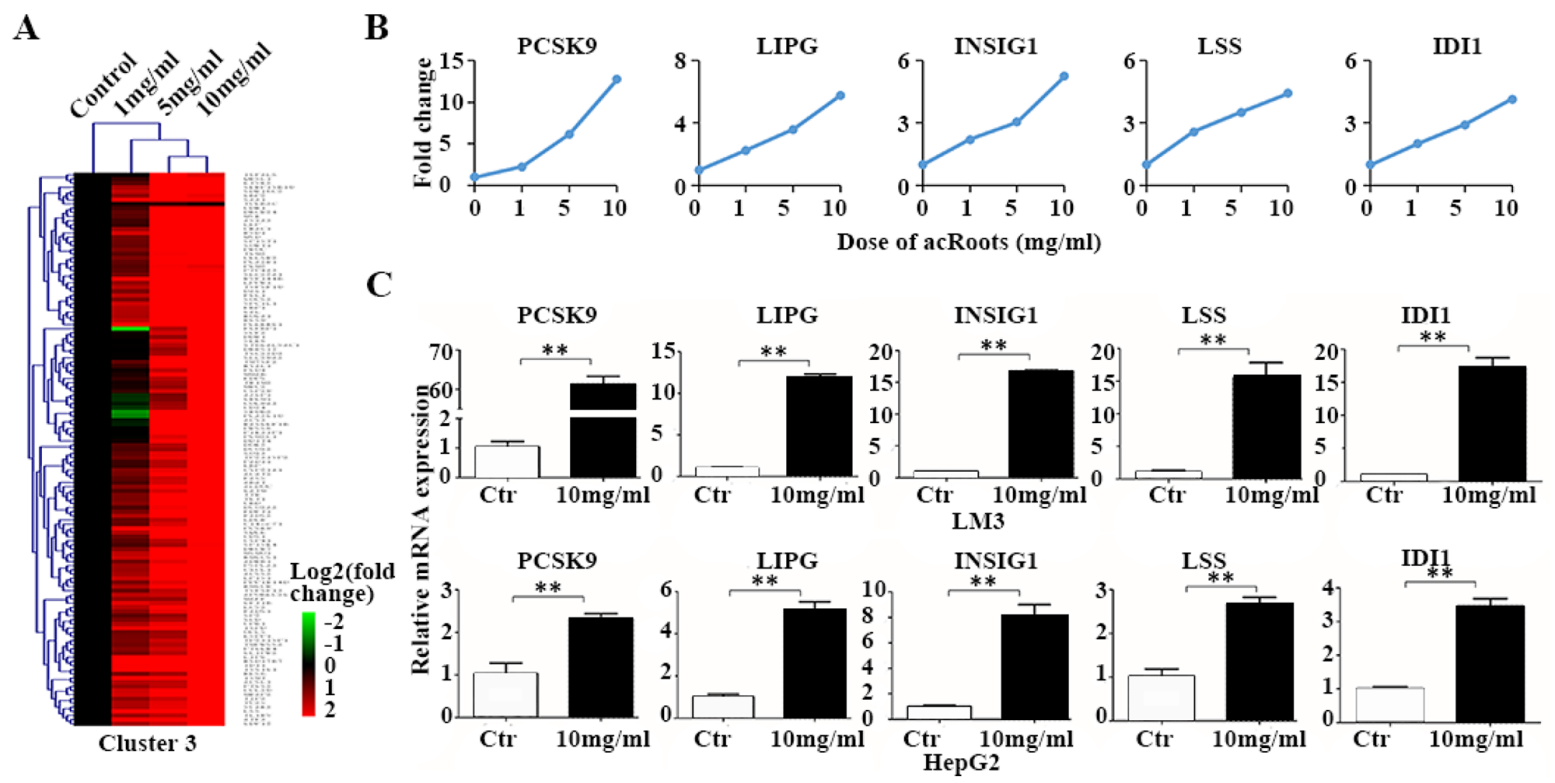
Metabolic genes up-regulated in LM3 cells by acRoots in a dose-dependent manner **A**. acRoots dose profiles for cluster 3; **B**. Example genes from cluster 3: five genes for which the mRNA array signal was > 6.0 and the flag was the p value (representing a higher expression); **C**. Validation of the metabolic gene profiles for the example genes by qRT-PCR in LM3 and HepG2 cells treated with acRoots. Data represent the mean ± SD of triplicate samples; **p < 0.05.

Of the eight clusters, clusters 2 and 3 (Supplementary Data sets 4 and 5) contained genes that were either up- or down-regulated by acRoots in a dose-dependent manner. We selected five genes from each of the two clusters that had a signal > 6.0 in the mRNA and the flag was the p value (representing a higher expression) to validate by quantitative reverse transcription PCR (qRT-PCR) (Figure 2 and Supplementary Figure 2). The results were consistent with those of the mRNA profiling, which validated the accuracy of the mRNA chip. Interestingly, *PCSK9* was the most significantly up-regulated metabolic gene by acRoots in LM3 cells (Figure 2B and 2C).

### Cholesterol signaling in LM3 cells treated with acRoots

**Figure 3:**
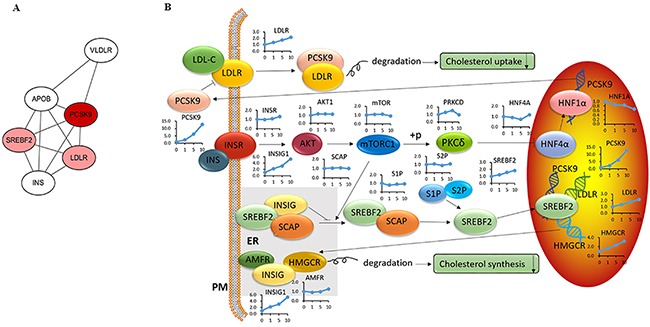
Modulation of the cholesterol signaling pathway in LM3 cells treated with acRoots **A**. PCSK9 molecular network; Red represents a close relationship; **B**. PCSK9 signaling pathway and associated changes in mRNA levels assessed by microarray analysis.

*PCSK9* is a secreted serine protease that regulates the post-transcriptional degradation of the low density lipoprotein (LDL) receptor (*LDLR*) [17] to reduce cholesterol uptake. To determine whether acRoots altered cholesterol metabolism in HCC cells in a *PCSK9*-dependent process, we analyzed the molecular network (www.string-db.org) and signaling pathways associated with *PCSK9* based on previous reports (Figure 3A and 3B) [18]. The corresponding changes in the mRNA level are shown next to the gene symbols (Figure 3B). Molecular network analysis indicated that sterol regulatory element binding transcription factor 2 (SREBF2) and *LDLR* were the closest links to PCSK9. Signaling pathway analysis revealed that *LDLR* and 3-hydroxy-3-methylglutaryl-CoA reductase (*HMGCR*), which are involved in cholesterol uptake and synthesis, respectively, were down-regulated in a dose-dependent manner, suggesting the possibility that acRoots inhibited cholesterol uptake and synthesis by altering *LDLR* and *HMGCR* expression in HCC.

### acRoots promotes *PCSK9* expression and inhibits cholesterol metabolism

**Figure 4:**
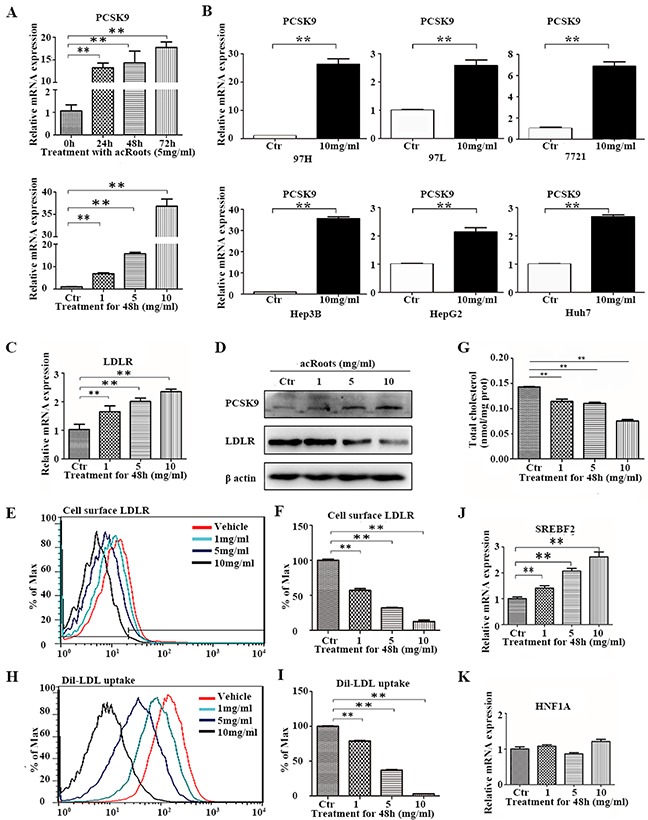
Alterations in PCSK9-related signaling pathways and cholesterol metabolism in LM3 cells in response to acRoots **A**. Changes in PCSK9 mRNA in LM3 cells treated with different doses of acRoots at the indicated time points; **B**. Increased PCSK9 expression in other HCC cell lines treated with acRoots; **C, D**. Detection of LDLR mRNA and protein expression. Inverse relationship between PCSK9 and total LDLR protein in acRoots-treated LM3 cells quantified by western blotting; **E, F**. Levels of cell surface LDLR expression quantified by flow cytometry analysis; **G, H**. Dil-LDL uptake analyzed by flow cytometry; **I**. Attenuation of the intracellular cholesterol level in complete medium by acRoots; **J, K**. Changes in the mRNA levels of transcription factors that regulate PCSK9 expression. Data represent the mean ± SD of triplicate samples; **p < 0.05.

To validate the results of the pathway analysis, we investigated the expression levels of the associated genes and cholesterol levels after treatment of HCC cells with acRoots. We determined that the level of *PCSK9* mRNA increased in acRoots-treated compared to untreated cells in a dose- and time-dependent manner (Figure 4A). Additionally, acRoots induced *PCSK9* expression in other HCC cell lines (Figure 4B). *LDLR* is a target of *PCSK9*, and previous studies have generally reported an inverse relationship with *PCSK9*. However, we did not observe a decrease in the *LDLR* mRNA level concomitant with up-regulation of *PCSK9* expression in acRoots-treated LM3 cells (Figure 4C). Therefore, we examined whether acRoots could alter the protein levels of total *LDLR* and/or cell surface *LDLR* expression. As predicted, acRoots treatment attenuated total and cell surface *LDLR* expression in a dose-dependent manner, indicating that acRoots-regulated *LDLR* reduction at the post-transcriptional level (Figures 4D, 4E, and 4F). We analyzed whether acRoots altered cholesterol metabolism as expected based on the pathway analysis. We evaluated 3,3′-dioctadecylindocarbocyanine-labeled low-density lipoprotein (Dil-LDL) uptake and the total cellular cholesterol level in LM3 cells. These data indicated that acRoots attenuated cholesterol uptake and intra-cellular cholesterol levels in a dose-dependent manner (Figures 4G, 4H, 4I, and Supplementary Figure 3).

Signaling pathway analysis revealed a potential upstream regulator *SREBF2* of acRoots-induced *PCSK9* expression. Previous studies have also demonstrated that the *SREBF2* and hepatocyte nuclear factor 1α (*HNF1A*) transcription factors bind to the *PCSK9* promoter and promote *PCSK9* transcription. Here, we showed that *SREBF2* was up-regulated in a acRoots dose-dependent manner, suggesting that acRoots promoted *PCSK9* expression by up-regulating *SREBF2* rather than *HNF1A* (Figure 4J and 4K).

### *PCSK9*-regulated signaling pathway promotes the effect of acRoots on HCC cells

**Figure 5:**
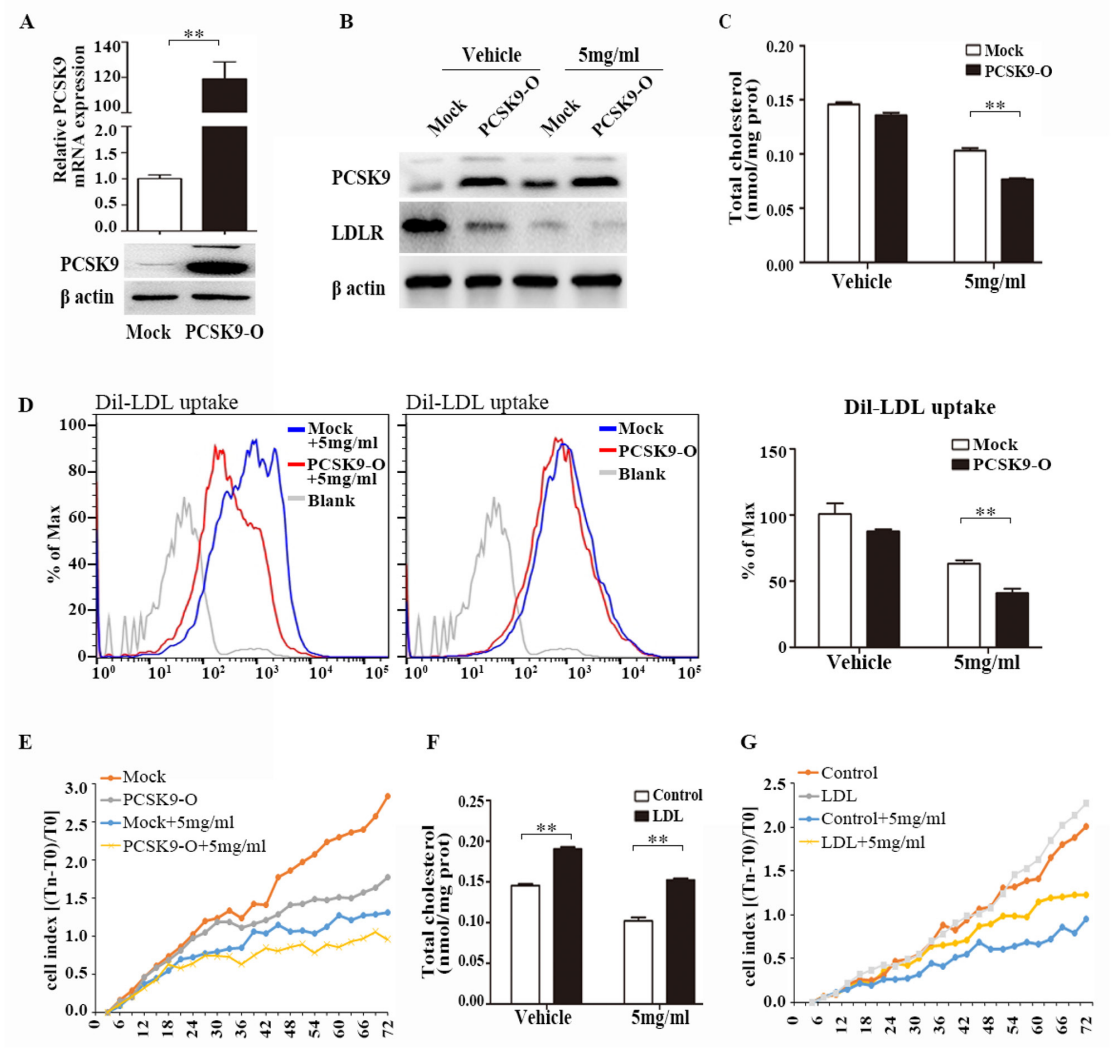
PCSK9 overexpression promotes the effect of acRoots Overexpression of PCSK9 in LM3 cells confirmed by qRT-PCR and western blot **A**. Forty-eight hours after transfection, LM3 cells were treated with acRoots (5mg/ml) for another forty-eight hours, then LDLR proteins expression **B**. total cholesterol concentration **C**. Dil-LDL uptake **D**. and cell viability **E**. were analyzed; LDL at 200ug/ml increases intracellular cholesterol contents **F**. LDL significantly rescued acRoots-reduced cell viability **G**. Data represent the mean ± SD of triplicate samples; **p < 0.05.

To determine whether *PCSK9* had a role in cholesterol metabolism and cell proliferation, we overexpressed *PCSK9* in LM3 cells. LM3-PCSK9 cells exhibited > 100-fold higher levels of *PCSK9* mRNA and protein (Figure 5A). Forty-eight hours after transfection, LM3 cells were treated with acRoots (5mg/ml) for another forty-eight hours, and then *LDLR* expression, Dil-LDL uptake, the total cholesterol concentration, and cell viability were assessed. We selected this concentration of acRoots because it was sufficient to induce *PCSK9* expression without obvious toxicity (Figure 1A, 4A, and Supplementary Figure 1A). Interestingly, *PCSK9* overexpression further decreased acRoots-mediated *LDLR* expression, Dil-LDL uptake, the total cholesterol concentration, and cell viability (Figure 5B, 5C, 5D and 5E).

Moreover, we detected the influence of cholesterol accumulation in acRoots-reduced cell viability by using human LDL, which containing 49% cholesterol, could increases intracellular cholesterol contents in HCC cells (Figure 5F). As shown in Figure 5G, LDL at 200ug/ml partially rescued acRoots-reduced cell viability (Figure 5G). These data suggested that *PCSK9*-regulated signaling pathway promotes the effect of acRoots on HCC cells.

### PCSK9 knockdown inhibits acRoots regulation of cholesterol metabolism

**Figure 6:**
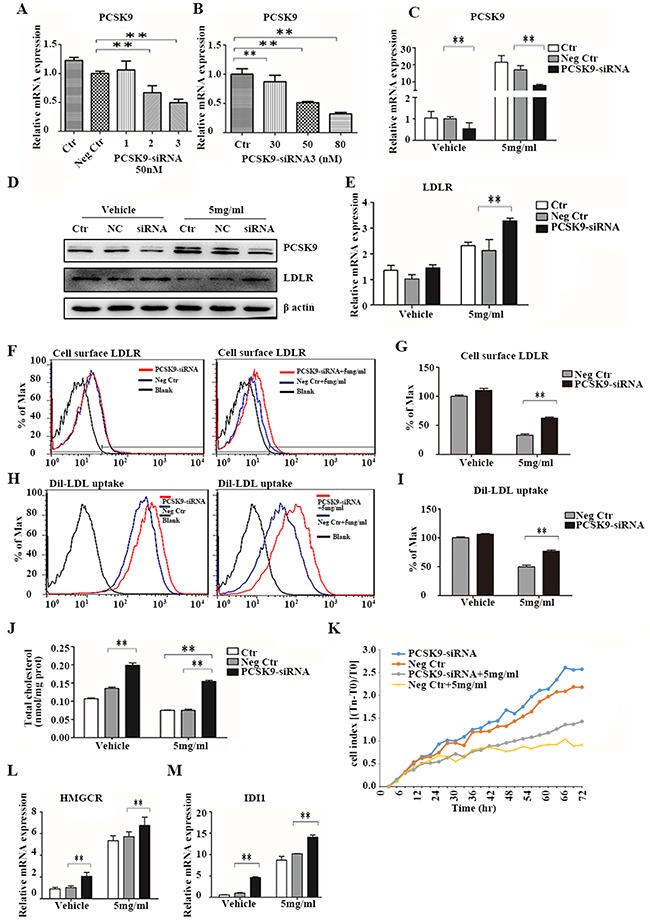
PCSK9 knockdown inhibits the effect of acRoots **A, B**. Identification of efficient PCSK9-siRNA sequences and measurement of the relative PCSK9 concentration 48 hours after transfection using qRT-PCR; **C-L**. LM3 cells transfected with PCSK9-siRNA for 48 hours were treated with acRoots (5 mg/mL) for an additional 48 hours and PCSK9 mRNA and protein, total LDLR **(C, D, E)**, and cell surface LDLR expression quantified **(F, G)**, Dil-LDL uptake (H, I), total cellular cholesterol levels **(J)**, cell proliferation **(K)**, mRNA levels of HMGCR **(L)**, and mRNA levels of IDI1 **M**. Data represent the mean ± SD of triplicate samples; **p < 0.05.

We next investigated whether *PCSK9* directly regulated cholesterol metabolism and cell proliferation in HCC cells in response to acRoots treatment, we knocked-down *PCSK9* in LM3 cells. The efficacy of *PCSK9* silencing by the selected siRNA was confirmed by real-time qRT-PCR (Figure 6A, 6B, and 6C). Forty-eight hours after transfection, LM3 cells were treated with acRoots (5 mg/mL) and *LDLR* expression, Dil-LDL uptake, total cellular cholesterol, and cell proliferation measured after 48 hours. As expected, the inhibition of total *LDLR* and surface *LDLR* protein expression by acRoots was significantly rescued by *PCSK9*-siRNA, while the *LDLR* mRNA level was still slightly up-regulated by acRoots (Figure 6D, 6E, 6F, and 6G). Additionally, knockdown of *PCSK9* ameliorated the effects of acRoots on Dil-LDL uptake, cellular cholesterol levels, and cell proliferation (Figures 6H, 6I, 6J, and 6K).

The intracellular cholesterol concentration depends on the rate of cholesterol uptake and synthesis. *PCSK9* knockdown improved cellular cholesterol uptake resulting in an increase in the total cellular cholesterol level. We investigated whether *PCSK9* knockdown also induced cholesterol synthesis. The mRNA levels of *IDI1* and *HMGCR*, which are involved in cholesterol synthesis, were analyzed by qRT-PCR. These results indicated that *PCSK9* knockdown resulted in an increase in *IDI1* and *HMGCR* levels in both acRoots-treated and untreated LM3 cells (Figure 6L and 6M). Thus, silencing of *PCSK9* increases cholesterol synthesis in HCC cells.

### The cholesterol-lowering effect of acRoots is independent of *INSIG1*

**Figure 7:**
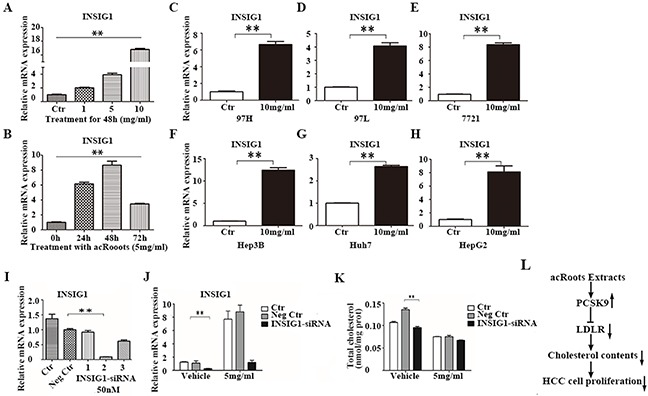
The cholesterol-lowering effect of acRoots is independent of INSIG1 **A, B**. Changes in PCSK9 mRNA levels in LM3 cells after treatment with the different doses of acRoots at the indicated time points; **C-H**. Increased INSIG1 expression in other HCC cell lines treated with acRoots; **I**. Identification of efficient INSIG1-siRNA sequences by qRT-PCR 48 hours after transfection; **J**. LM3 cells transfected with PCSK9-siRNA for 48 hours were treated with acRoots (5 mg/mL) for an additional 48 hours and the relative mRNA levels of INSIG1 quantified; **K**. LM3 cells transfected with PCSK9-siRNA for 48 hours were treated with acRoots (5 mg/mL) for an additional 48 hours to quantify the relative levels of total cellular cholesterol; **L**. Schematic representation of the working model. Data represent the mean ± SD of triplicate samples; **p < 0.05.

*INSIG1* expression was altered after treatment of LM3 cells with acRoots (Figure 2B and 3B). *INSIG1* expression was up-regulated by acRoots in a dose- but not time-dependent manner (Figure 7A and 7B). *INSIG1* expression was also increased in response to acRoots treatment in other HCC cell lines (Figure 7C, 7D, 7E, 7F, 7G, and 7H). The expression of *INSIG1* is induced by insulin. *INSIG1* encodes an endoplasmic reticulum membrane protein that regulates cholesterol metabolism. This protein may act as a scaffold to recruit the reductase that stimulates ubiquitination and proteasomal degradation of *HMGCR*. This results in inhibition of cholesterol synthesis because *HMGCR* is the rate-limiting enzyme of this pathway. We hypothesized that the cholesterol-lowering effect of acRoots could result from up-regulation of *INSIG1*. To test this hypothesis, we silenced *INSIG1* in LM3 cells. The efficacy of siRNA-mediated silencing was confirmed by real-time qRT-PCR (Figure 7I and 7J). Forty-eight hours after transfection with the selected *INSIG1*-siRNA, LM3 cells were treated with acRoots (5 mg/mL) and the total intracellular cholesterol level analyzed after 48 hours (Figure 7K). These data indicated that *INSIG1* knockdown did not rescue the cholesterol-lowering effect of acRoots.

## DISCUSSION

Metabolic alterations are a hallmark of cancer cells. The most prominent metabolic alterations are a switch to glycolysis in the presence of oxygen [19] and *de novo* lipid synthesis [20, 21]. These pathways produce the nutrients to support uncontrolled cancer cell proliferation. Although our understanding of the metabolic alterations that drive cancer progression has improved, there is still a lack of effective therapeutic strategies. The traditional Chinese medicine acRoots is widely used as crude drug to treat various cancers [22] including liver, lung, and breast cancer. However, the mechanisms underlying the anti-tumor effects of acRoots have not been elucidated. In this study, we demonstrated that acRoots reduces HCC cell viability, and inhibits cholesterol uptake and synthesis at a dose that is not toxic to normal liver cells. These data suggest that acRoots has anti-tumor efficacy.

Cholesterol has a critical role in multiple cellular functions including membrane synthesis, steroid synthesis, lipid raft formation, maintenance of membrane fluidity, and transmembrane signaling [23]. Intracellular cholesterol is generated by dietary uptake through the *LDLR* and from endogenous cholesterol biosynthesis [24]. In normal mammalian cells, cholesterol metabolism is maintained at a steady level through complex regulatory mechanisms. However, the strong requirement for cholesterol to support the growth and proliferation of malignant cells results in increased cholesterol synthesis and uptake [25, 26]. We demonstrated that acRoots inhibited cholesterol metabolism and proliferation in LM3 cells in a dose-dependent manner, suggesting that acRoots may suppress tumor growth by lowering cholesterol levels. KEGG pathway analysis also revealed that the mRNA levels of several metabolic genes involved in steroid synthesis and terpenoid backbone biosynthesis were up-regulated in response to acRoots treatment in a dose-dependent manner (Supplementary Figure 1C and 1D). These results suggest acRoots inhibits intracellular cholesterol levels through upregulation of *PCSK9*, which in turn induces a metabolic feedback loop in LM3 cells.

*PCSK9* is a secreted serine protease that is involved in the post-transcriptional regulation of LDL (promotes intracellular degradation in acidic subcellular compartments) [27]. *LDLR* is the receptor for serum LDL. Therefore, *PCSK9*-induced degradation of *LDLR* results in reduced intracellular cholesterol uptake and increased serum LDL-cholesterol levels [28, 29]. Decreased *PCSK9* expression and increased *LDLR* expression have been observed in HCC tissues, which may provide a constant supply of cholesterol in the HCC microenvironment [30]. Here, we demonstrated that acRoots promotes *PCSK9* expression and inhibits *LDLR* expression at the post-translational level. *PCSK9* knockdown rescued the cholesterol-lowering and anti-proliferative effects of acRoots, suggesting that *PCSK9* has a key role in cholesterol metabolism and that *PCSK9* and *LDLR* expression are inversely correlated.

*SREBF2*, a membrane-bound transcription factor, regulates cholesterol homeostasis [31]. *SREBF2* is activated in response to reduced cholesterol levels and up-regulates the expression of genes responsible for cholesterol synthesis and uptake. *SREBF2* activates *PCSK9* and *LDLR* transcription through binding to functional sterol regulatory elements in the promoters of these genes [32]. *HNF1A*, a liver-enriched basal transcription factor, cooperates with *SREBF2* to promote *PCSK9* transcription [33]. Insulin stimulation of mammalian target of rapamycin complex 1 (*mTORC1*) was previously shown to initiate a signaling cascade that results in nuclear exclusion of *HNF1A* and decreased *PCSK9* transcription. Conversely, inhibition of hepatic *mTORC1* in the mouse liver by rapamycin or insulin receptor knockdown stimulates *PCSK9* expression and reduces *LDLR* levels [18]. We observed no changes in the expression of proteins in the insulin*/mTORC1/HNF1A* pathway in LM3 cells after treatment with acRoots. However, we did observe dose-dependent upregulation of *SREBF2* expression in response to acRoots treatment, suggesting that *SREBF2* is the upstream transcription factor that regulates acRoots-induced expression of *PCSK9*.

*INSIG1* is also up-regulated by acRoots. The *INSIG1* protein recruits the reductase that promotes degradation of *HMGCR* and inhibits cholesterol synthesis [34]. In the present study, acRoots treatment resulted in decreased total cholesterol levels. Therefore, we hypothesized that the cholesterol-lowering effect of acRoots resulted from the inhibition of cholesterol uptake by *PCSK9* and cholesterol synthesis by *INSIG1*. However, our data indicated that *INSIG1* knockdown failed to rescue the cholesterol-lowering effect of acRoots.

acRoots extract consists of many compounds. According to previous reports, the inhibition of cell viability might be a result of multiple components, including triterpenes, flavonoids and phenolics [10, 11]. In the present study, we first demonstrated the role of acRoots in cholesterol metabolism. However, the concrete components involved in regulating cholesterol metabolism are still unknown. Further isolation and identification of the bioactive components would be conducted in our subsequent work.

In conclusion, our data indicate that acRoots significantly enhances *PCSK9* expression, which decreases *LDLR* expression at the post-transcriptional level, inhibits LDL uptake, lowers the total intracellular cholesterol concentration, and suppresses cell viablity in LM3 cells (Figure 7L). Additionally, silencing of *PCSK9*, but not *INSIG1*, attenuates the cholesterol-lowering effect of acRoots, suggesting that *PCSK9* has a key role in acRoots-regulated cholesterol metabolism in HCC cells. The effects of acRoots on cholesterol metabolism must be further analyzed *in vivo* to validate the anti-tumor efficacy.

## MATERIALS AND METHODS

### Cell lines and cell culture

The HCCLM3, MHCC97H, and MHCC97L cell lines were established at the Liver Cancer Institute of Fudan University. HepG2, SMMC-7721, Huh7, Hep3B, and HL-7702 cells were purchased from the Cell Bank of the Institute of Biochemistry and Cell Biology, China Academy of Sciences, Shanghai, China. Cells were cultured in DMEM with high glucose (Gibco BRL, NY, USA) supplemented with 10% fetal bovine serum (Gibco BRL, NY, USA). All cells were incubated at 37°C in a humidified atmosphere containing 5% CO_2_.

### Preparation of acRoots

*Actinidia chinensis* Planch roots were chopped and immersed in ten volumes of double distilled water. The mixture was then heated to 100°C and incubated for 1 hour. Finally, the decoction was filtered. The drug was obtained after two cycles of this procedure and was concentrated to generate a 1 g/mL working solution.

### Cell viability and proliferation assays

HCC cell viability was assessed using CCK-8 assays (Cell Counting Kit-8, Dojindo, Japan). Briefly, 4,000 cells were seeded in 96-well culture plates. The cells were allowed to attach for 6 hours. We then added 100 mL CCK-8 assay buffer to each well after 0, 24, 48, and 72 hours. The optical density (450 nm) was measured in each well using a microplate reader (BIO-TEK, Winooski, VT, USA) according to the manufacturer's instructions. Three independent experiments were performed.

Changes in cell proliferation were recorded using Cell-IQ, an integrated, fully automated system (Chip-Man Technologies Ltd., Finland) as described previously [35]. LM3 cells were plated onto 24-well plates at an appropriate density. The system automatically selected three or four visual fields in each well and recorded images every 30 minutes for 4 days. The proliferation rate was analyzed with the Cell-IQ Analyser Pro-Write V.AN 2.3.0 (Chip-Man Technologies Ltd.).

### Gene expression profiling

LM3 cells were treated with acRoots (0, 1, 5, and 10 mg/mL concentrations) for 72 hours and total RNA extracted using the TRIzol Reagent (Invitrogen, Carlsbad, CA, USA). Total RNA was amplified and labeled using the One-color Low Input Quick Amp Labeling Kit (Agilent Technologies, Santa Clara, CA, USA), according to the manufacturer's instructions. Labeled cRNA was purified using the RNeasy Mini Kit (Qiagen, Hilden, Germany). We then hybridized 1.65 μg Cy3-labeled cRNA to the arrays using the Agilent Gene Expression Hybridization Kit (Agilent) in a hybridization oven (Agilent) for 17 hours. Following the incubation, the slides were washed in staining dishes (Thermo Scientific, Waltham, MA, USA) with the Gene Expression Wash Buffer (Agilent) and scanned on an Agilent microarray scanner using the default settings. Data were extracted using the Feature Extraction software version 10.7 (Agilent). Raw data were normalized using a quantile algorithm and the Gene Spring software version 11.0 (Agilent).

### Gene set annotation by metabolic process

In order to identify all metabolic genes regulated by acRoots in LM3 cells, we developed a filtering strategy based on the following criteria: a) All metabolism-related genes must have been annotated to the term “metabolic process” (GO: 0008152) or its children in Gene Ontology (http://amigo1.geneontology.org/) (Supplementary Data set 1 and 2); and b) The metabolic genes regulated by acRoots in LM3 cells were required to exhibit at least a two-fold change in response to any concentration of acRoots relative to the untreated control group (Supplementary Data set 3). A correlational formula (VLOOKUP) was used to correlate the data from Supplementary Data set 1 with that of Supplementary Data set 4 and identify all metabolic genes regulated by acRoots in LM3 cells (Supplementary Data set 3).

### Pathway analysis

The DAVID software was used to determine KEGG pathway enrichment. All metabolic genes regulated by acRoots in LM3 cells were queried in the DAVID database. Pathway information was downloaded from the KEGG database (Supplementary Data set 6).

### RNA isolation and real-time qRT-PCR analysis

Total RNA was isolated from LM3 cells with the TRIzol reagent (Invitrogen) according to the manufacturer's protocol. Complementary DNA was synthesized from 500 ng RNA using the Reverse Transcription Reagent Kit (TaKaRa, Shiga, Japan). All primers are shown in Supplementary Table 1. Real-time qRT-PCR analysis was performed using an Applied Biosystems 7500 Real-Time PCR System. Values were derived from at least three independent experiments performed in duplicate and normalized to β-actin expression. The2^−△△*Ct*^ method was used for the data analysis [36].

### Western blot analysis

Western blotting was performed as described [37]. The primary antibodies (anti-PCSK9, and anti-LDLR) were purchased from Abcam (Cambridge, MA, USA). The anti-β-actin antibody was obtained from Santa Cruz Biotechnology Inc. (Santa Cruz, CA, USA). The secondary antibodies were either anti-mouse or anti-rabbit HRP-linked. Blots were developed using the Pierce ECL Western Blotting Substrate (Pierce Biotechnology, IL, USA).

### Cell transfection

Gene silencing was performed by transfection LM3 cells with siRNA oligonucleotides (GenePharma, Shanghai, China). All siRNA sequences are shown in Supplementary Table 2. For transfections in 12-well plates, 1.0 × 10^5^ cells were seeded per well and the cells transfected using the siPORT^TM^*NeoFX*^TM^ Transfection Agent (Invitrogen) according to the manufacturer's protocol. Efficient siRNA oligonucleotides were detected by qRT-PCR. *PCSK9* overexpression in LM3 cells was performed by transfecting the cells with the indicated PCSK9 constructs (GenePharma) using Lipofectamine 2000 (Invitrogen) according to the manufacturer's instructions. Triplicate samples were analyzed in all experiments and all experiments were repeated at least three times.

### Flow cytometry

Cell surface *LDLR* was detected by flow cytometry. Following treatment, LM3 cells were detached from the plates by scraping and washed with phosphate-buffered saline (PBS) containing 5% bovine serum albumin for 30 minutes at room temperature. Cells were then washed with PBS, incubated with an anti-LDLR antibody (Abcam) at room temperature for 1 hour, and then incubated with an Alexa Fluor 488-conjugated goat anti-rabbit IgG secondary antibody (Abcam) for 30 minutes at room temperature. Background fluorescence (control) was quantified using cells incubated with an isotype rabbit IgG antibody (Abcam) for 1 hour and Alexa Fluor 488-conjugated goat anti-rabbit IgG for 30 minutes. The cells were washed and resuspended in PBS, and then analyzed by flow cytometry using an FL1 emission filter (FACScan, BD Biosciences, San Jose, CA, USA). The data were analyzed using the FlowJo software.

Dil-LDL (Biomedical Technologies Inc., USA) uptake was representative of total cellular cholesterol uptake. Following treatment of LM3 cells, the media was exchanged to serum-free and incubated with 10 ug/mL Dil-LDL at 37°C for 12 hours. The cells were detached with trypsin, washed, and the resuspended in PBS for flow cytometry analysis using an FL2 emission filter (FACScan, BD Biosciences, San Jose, CA, USA). Data were analyzed using the FlowJo software (CA, USA).

### LDL preparation

LDL was purchased from AngYu Biotechnologies (Shanghai, China). It is isolated from blood bank produced human plasma. It is purified via ultracentrifugation to homogeneity determined by agarose gel electrophoresis. Each lot is analyzed on agarose gel electrophoresis for migration versus LDL.

### Measurement of total cellular cholesterol

Total cellular cholesterol was measured with the Cholesterol Quantitation Kit from Applygen (Peking, China) according to the manufacturer's protocol. The activities of cholesterol esterase and cholesterol oxidase, and the amount of H_2_O_2_ produced were quantified using this colorimetric assay. The values were normalized against the total protein concentration in each sample, which was estimated using Bicinchoninic Acid assays (Beyotime Biotechnology, China).

### Statistical analysis

Statistical analysis was performed using the SPSS16.0 statistical software (SPSS Inc, Chicago, IL, USA). Student's *t*-tests were used to assess significant differences between the study groups. The level of significance was p < 0.05. All experiments were performed at least three times.

## SUPPLEMENTARY FIGURES, TABLES AND DATA SETS














