# Supplementary material for: Actinidia chinensis Planch root extract inhibits cholesterol metabolism in hepatocellular carcinoma through upregulation of PCSK9
Source: Oncotarget. 2017 Feb 2;8(26):42136–48. doi: 10.18632/oncotarget.15010 (PMC5522055; doi:10.18632/oncotarget.15010)
Supplement: Supplementary file 1 [file oncotarget-08-42136-s001.pdf]

Actinidia chinensis Planch root extract inhibits cholesterol metabolism in hepatocellular carcinoma through upregulation of *PCSK9*

SUPPLEMENTARY MATERIALS

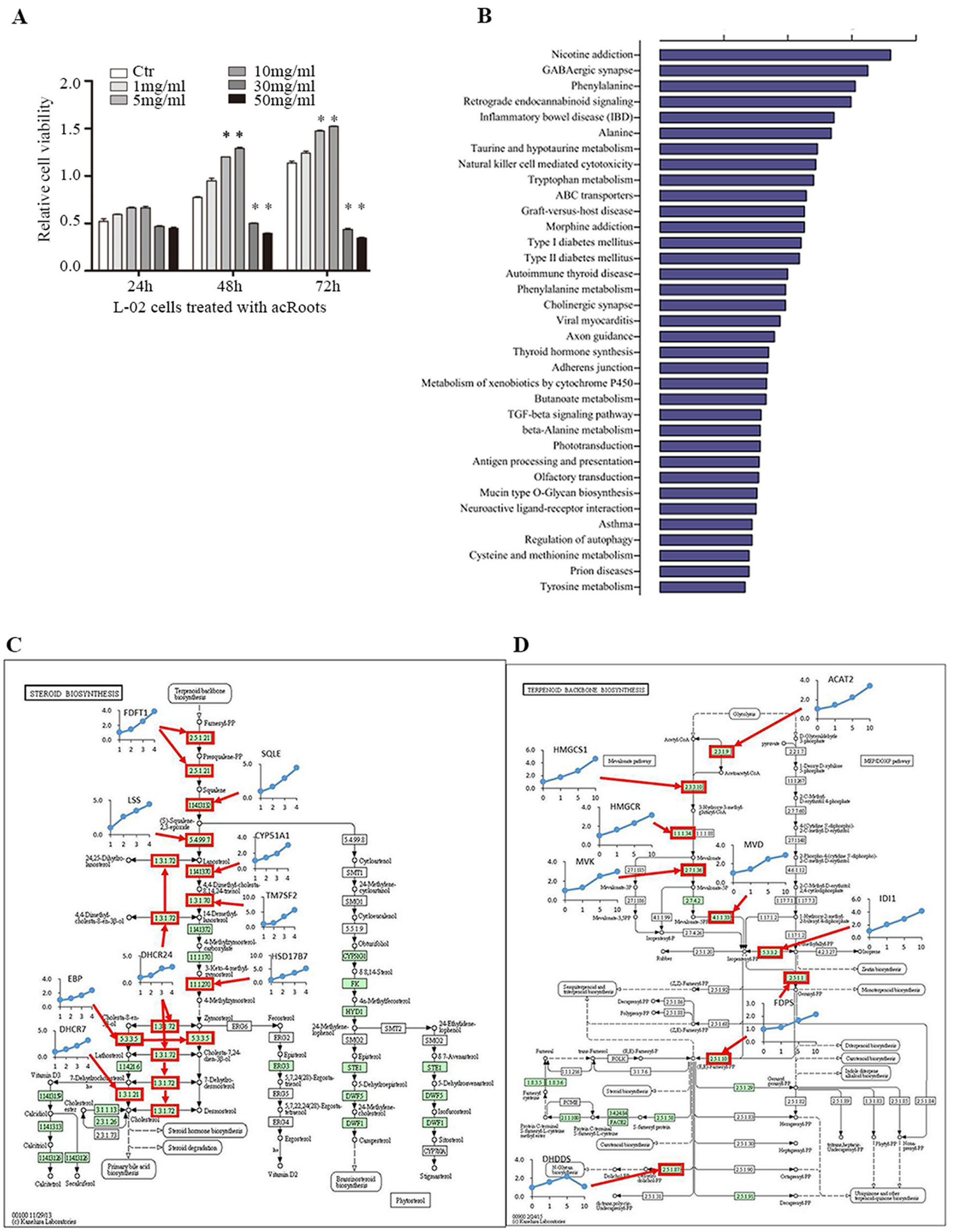

Supplementary Figure 1: A. The responses of the indicated signaling pathways to acRoots in LM3 cells; **B, C.** Map of the steroid and terpenoid backbone biosynthesis signaling pathways from KEGG. The corresponding changes in the mRNA levels of each gene in the signaling pathway are shown next to the gene symbol.

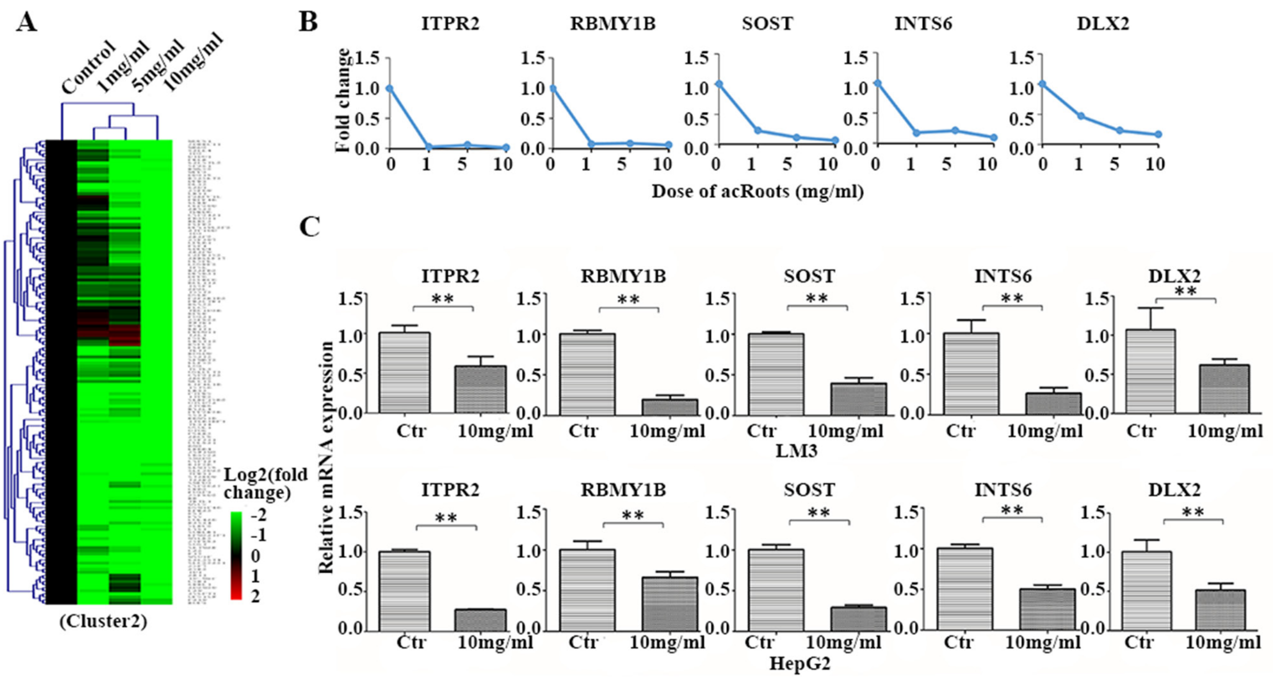

Supplementary Figure 2: Metabolic genes down-regulated in LM3 cells after acRoots treatment in a dose-dependent manner. A. Dose profiles for cluster 2; **B.** Example genes from cluster 2: Five genes for which the signal was > 6 in the mRNA array and the flag was P (representing a higher expression); **C.** Validation of the metabolic gene profiles by qRT-PCR of example genes in acRoots-treated LM3 and HepG2 cells.

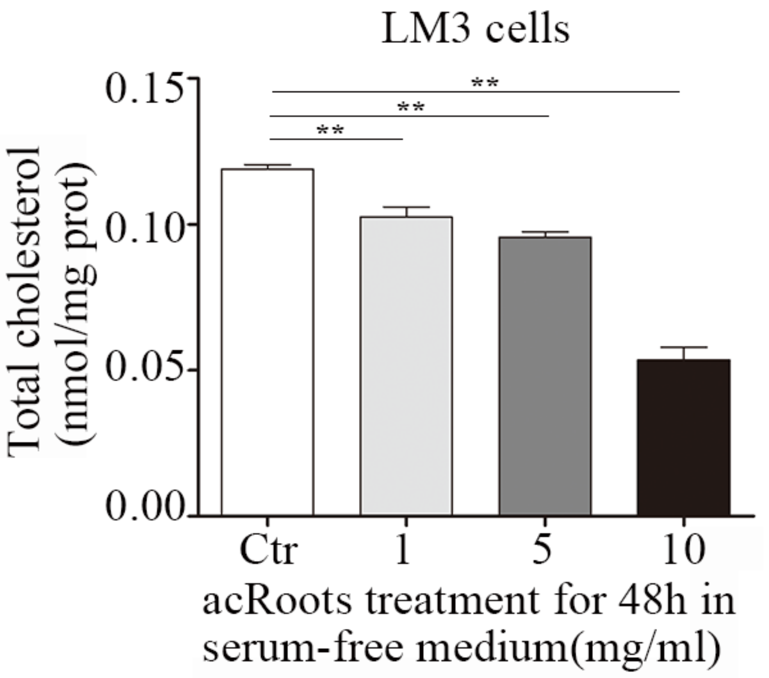

Supplementary Figure 3: Attenuation of intracellular cholesterol levels in serum-free media in response to acRoots treatment.

Supplementary Table 1: Primers for PCR used in this study

Gene	Forward (5’→3’)	Reverse (5’→3’)
PCSK9	AGACCCACCTCTCGCAGTC	GGAGTCCTCCTCGATGTAGTC
LDLR	GCTACCCCTCGAGACAGATG	CACTGTCCGAAGCCTGTTCT
SREBF2	GGTTGTCGGGTGTCATGGG	TTGCAGCATCTCGTCGATGT
HMGCR	GAGCGTGCGTAAGGTGAGG	ACAGAATCCTTGGATCCTCCAG
HNF1A	ATTTGTTCCCAAGAGCATCA	CCCACAGGAGTAAGGACGAC
LSS	CCTGTGCCATCTCCTACACG	TTGAAGGCCATGGAACGCA
LIPG	AGGATGAGCAACTCCGTTCC	TGGAGCTTATCTTCCAGCCG
IDI1	CGGAGGCTGATCAGTGTTCTA	TCTCTGCCAGGAGTTGAACC
ITGB2	TCAGGACTTTACGACCCGC	ACTCCTGAGAGAGGACGCA
INSIG1	ATCTTTTCCTCCGCCTGGTG	CCAATTTAGCACTGGCGTGG
ITPR2	GCGCAGGGAAGAAGAGGGA	GTCATCCACTAACCCCAAGGT
DXL2	CACCTCCTACGCTCCCTATG	TTGGCTTCCCGTTCACTATC
INTS6	AGCCGCCCTATGCTATCAAG	CAAGAGTCGTAAGTCCTTCAGC
SOST	ACACAGCCTTCCGTGTAGTG	GGTTCATGGTCTTGTTGTTCTCC
β-actin	CGTGGACATCCGTAAAGACC	ACATCTGCTGGAAGGTGGAC

Supplementary Table 2: siRNA sequences for gene silencing used in this study

siRNA	siRNA Sequence (5’-3’)
PCSK9-siRNA-1	Sense: CCAAGAUCCUGCAUGUCUUTT
	Antisense: AAGACAUGCAGGAUCUUGGTT
PCSK9-siRNA-2	Sense: GGUGGAGGUGUAUCUCCUATT
	Antisense: UAGGAGAUACACCUCCACCTT
PCSK9-siRNA-3	Sense: CCUCAUAGGCCUGGAGUUUTT
	Antisense: AAACUCCAGGCCUAUGAGGTT
INSIG1-siRNA-1	Sense: GGUGAGCACAAUGUAUUCATT
	Antisense: UGAAUACAUUGUGCUCACCTT
INSIG1-siRNA-2	Sense: CGCAGUUUCUCGUGUAUAATT
	Antisense: UUAUACACGAGAAACUGCGTT
INSIG1-siRNA-3	Sense: GACUUUAGCAGCCCUAUCUTT
	Antisense: AGAUAGGGCUGCRAAAGUCTT
Negative Control	Sense: UUCUCCGAACGUGUCACGUTT
	Antisense: ACGUGACACGUUCGGAGAATT

Supplementary Data set 1: All metabolism-related genes annotated by metabolic process (GO:0008152) and its children in Gene Ontology.

See Supplementary File 1

Supplementary Data set 2: All genes regulated by acRoots in LM3 cells were at least 2-fold change caused by any drug concentration group compared to untreated group.

See Supplementary File 2

Supplementary Data set 3: All metabolic genes regulated by acRoots in LM3 cells were at least 2-fold change caused by any drug concentration group compared to untreated group.

See Supplementary File 3

Supplementary Data set 4: Cluster 2 - fold change.

See Supplementary File 4

Supplementary Data set 5: Cluster 3 - fold change.

See Supplementary File 5

Supplementary Data set 6: KEGG_PATHWAY.

See Supplementary File 6
